# Desmoid Tumour of the Brachial Plexus

**DOI:** 10.1155/2013/575982

**Published:** 2013-06-12

**Authors:** Orege Juliette, Koech Florentius, Ndiangui Francis, Benson Ndegwa Macharia, Mbaruku Neema

**Affiliations:** ^1^Department of Radiology, Moi University School of Medicine, P.O. Box 4606, Eldoret 30100, Kenya; ^2^Department of Neurosurgery, Moi University School of Medicine, P.O. Box 4606, Eldoret 30100, Kenya; ^3^Department of Pathology, Moi Teaching and Referral Hospital, P.O. Box 3, Eldoret 30100, Kenya; ^4^Department of Pathology, Moi University School of Medicine, P.O. Box 4606, Eldoret 30100, Kenya; ^5^Department of Orthopedics, Moi University School of Medicine, P.O. Box 4606, Eldoret 30100, Kenya

## Abstract

Desmoid tumours of the brachial plexus are rare and may occur in extra-abdominal sites. The tumours are of fibroblastic origin and, although benign, are locally aggressive. Their relationship to critical neurovascular structures in their anatomic locations presents a challenge to the operating surgeons trying to adhere to the principles of surgery. Surprisingly little neurosurgical literature exists which was devoted to this topic despite the challenge these lesions present in surgery both at surgery and in choosing adjuvant therapies. We report a case of a large brachial plexus tumour in a patient which was diagnosed radiologically and histopathologically and the patient underwent surgical excision with good outcome. Desmoid tumours histologically are benign and are usually composed of proliferating, benign fibroblasts in an abundant matrix of collagen. They do not transform into malignant tumours or metastasize. Surgery is the mainstay of treatment; however, adjuvant radiation and chemotherapy remain controversial.

## 1. Introduction

Many terms have been used to refer to desmoids tumours over the years, including fibromatosis, desmoid tumors, and aggressive fibromatosis. However, “Desmoid-type fibromatosis” has emerged as the designation of choice by the World Health Organization [1]. 

A review of the literature identified three case series reporting the treatment of desmoids tumours involving the brachial plexus. The first series, reported by Binder et al. [2] in June 2004, served to ascertain the rarity of these tumours. Twenty-four patients were treated at the University of California, San Francisco, CA, USA, who had primary brachial plexus tumours and only one (4%) had a desmoid tumour. The second case series reported by Seinfeld et al. [3] in 2006 included four cases of desmoid-type fibromatosis involving the brachial plexus. This series additionally assessed these lesions for mutations in the *c-KIT *oncogene in hopes of establishing a basis for predicting which of these lesions would respond to the chemotherapy agent imatinib mesylate. In the third case series, Dafford et al. [4] in June 2007 undertook a retrospective study of 15 desmoid tumors in 11 women and four men (ranging in age from 32 to 67 years; median 48 years) treated at their institution. In this study, the results were that there were 13 patients (86%) with brachial plexus lesions. 

In this review, we document the clinical presentation, neuroimaging, surgical, and pathological findings in a patient with a desmoid tumour arising from the brachial plexus.

### 1.1. Age and Gender

Incidences of desmoids tumours are low and range from 0.2 to 0.4 per 100,000 with two peaks in incidence among individuals aged 6 years to 15 years and between puberty and the age of 40 in women [1, 5]. A Finnish study showed the age distribution profile to demonstrate four distinct peak periods: the juvenile period, the fertile period, the middle-age period, and the old-age period [6]. The juvenile desmoid tumor is an extra-abdominal tumor in young girls, the fertile type is an abdominal tumor in women, and the middle age kind is also abdominal but the sex ratio is almost equal, whereas in the old age group, both abdominal and extra-abdominal tumors are frequent and equal in both sexes [5]. A low growth rate has been recorded in men and young girls unlike the fast growth rate in women.

### 1.2. Treatment

The mainstay of treatment is currently surgical resection with wide margins [7]. For recurrent or inoperable disease, the inclusion of radiotherapy and cytotoxic and noncytotoxic (hormonal) chemotherapy remains controversial [8]. 

### 1.3. Histology

On histology, desmoid tumour is a proliferation of cytologically benign-appearing fibrocytes [9]. 

### 1.4. Outcome

These tumours can recur, but with surgery and postoperative radiotherapy, the progression-free survival was 95% at 5 years and 93% at 10 years [10]. 

## 2. Case Report

**Figure 1:**
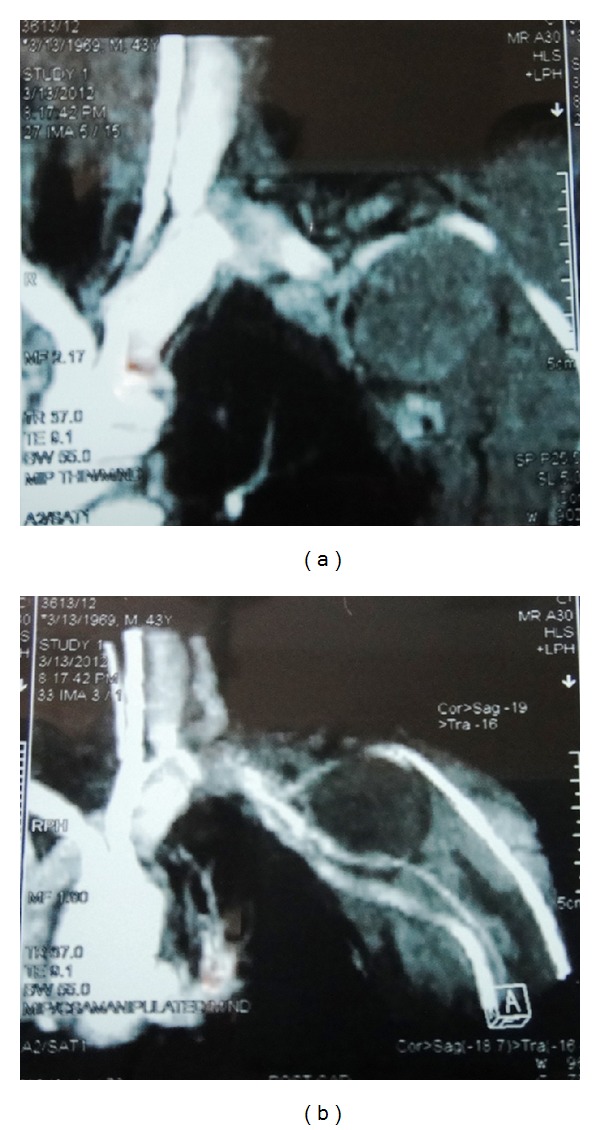
Coronal MRI T1W with gadolinium (a) and (b) images showing a well-defined brachial plexus desmoid tumour that was isointense to muscle on T1W sequences and mixed signal intensity (both low to high signal intensity) on T2W sequences and was straddling the brachial plexus.

**Figure 2:**
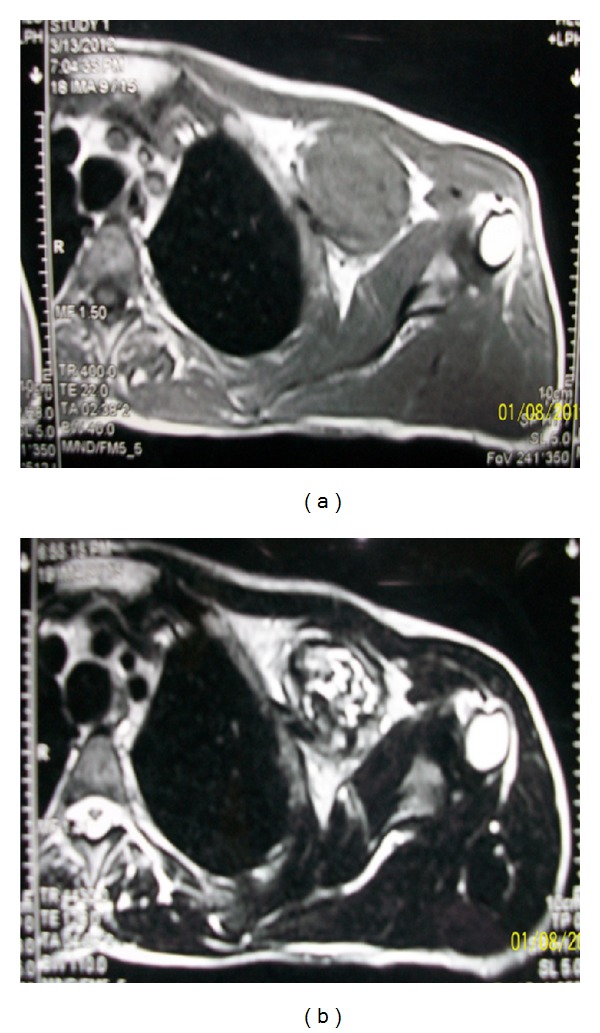
Axial MRI T1W (a) and T2W (b) images showing a well-defined brachial plexus desmoids tumour that was isointense to muscle on T1W sequences and mixed signal intensity (both low to high signal intensity) on T2W sequences and was straddling the brachial plexus.

**Figure 3:**
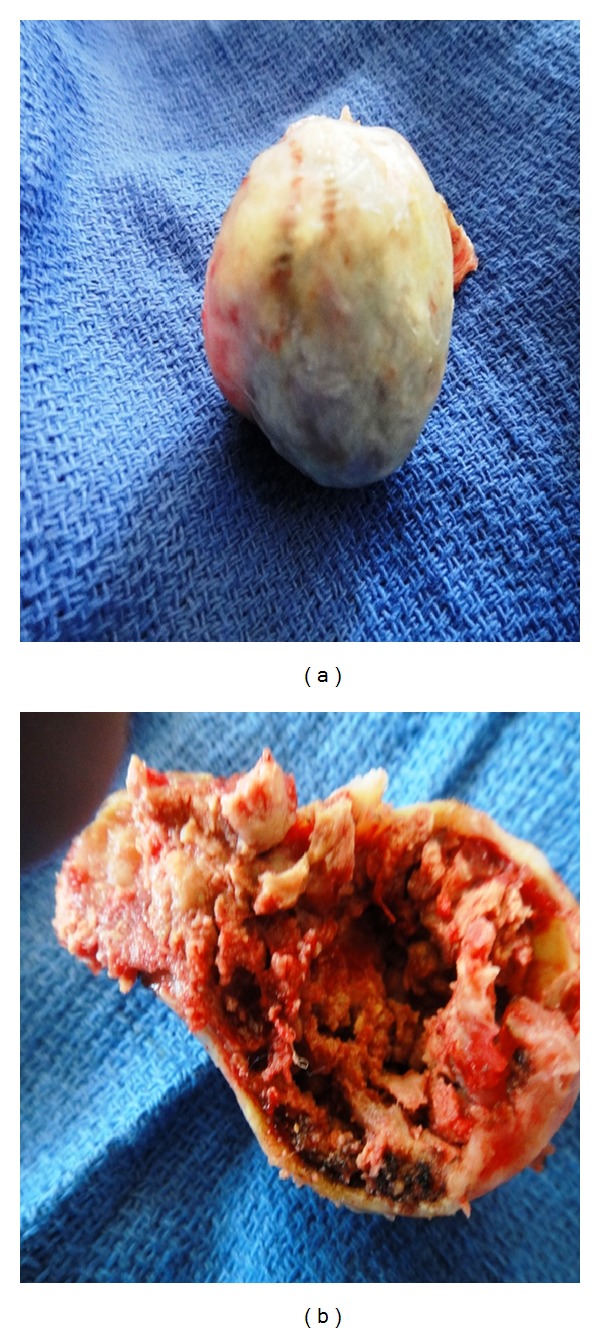
Gross specimens of the desmoid tumour (a) and (b).

**Figure 4:**
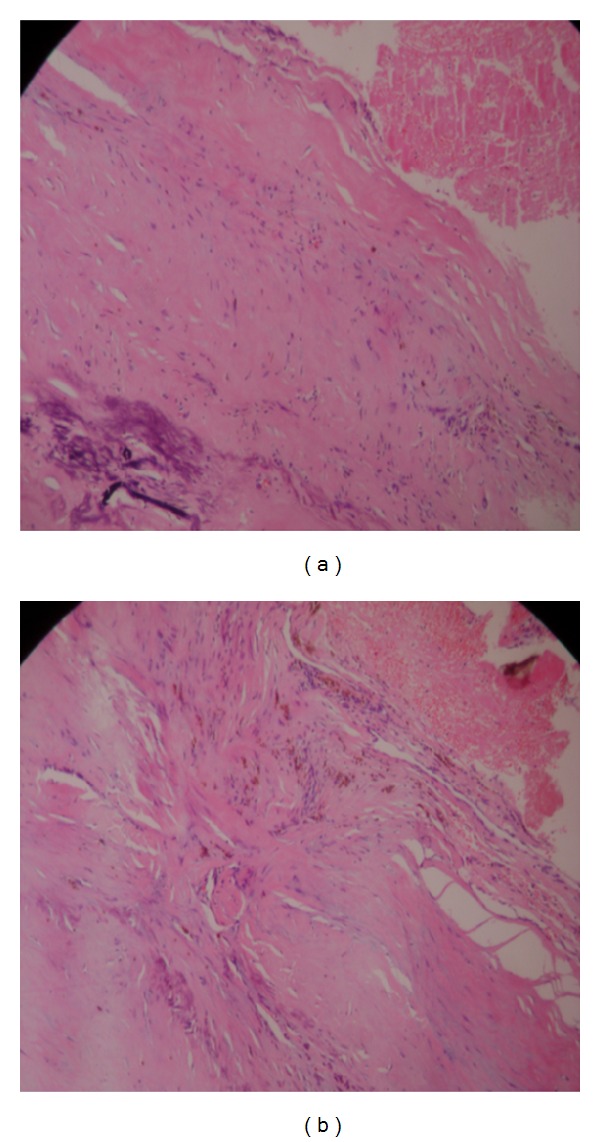
Histology showing a desmoid tumour.

A 43-year-old man presented with a 10-year history of a left outer infraclavicular mass which was slow in progression. He also had difficulty in flexing the left arm, numbness, and pain in the left middle and index finger. Examination revealed a large mass in the outer 1/3 below the left clavicle. The mass was firm, fixed, and tender on palpation. Flexion movements at the elbow joint were limited. The brachial and radial pulses were palpable. There were no similar lesions or nodules elsewhere in the body. An MRI of the neck including the thoracic inlet (Figures 1(a), 1(b), 2(a), and 2(b)) revealed a well-defined brachial plexus mass that was isointense to muscle on T1W sequences and mixed signal intensity (both low to high signal intensity) on T2W sequences and was straddling the brachial plexus. The patient was seen in the outpatient neurosurgical clinic. The patient was admitted and prepared for excision of the tumour. At surgery through a linear incision over the outer 1/3 infraclavicular, the skin and the muscle layers were reflected. Through microsurgical technique, the tumour was identified between the lateral and medial cords of the brachial plexus. Gentle dissection and isolation of brachial plexus cords were done, securing safely the supraclavicular vessels and the dome of the lung. Total macroscopic excision was achieved (Figures 3(a) and 3(b)). Histological examination revealed a fibrous wall covering amorphous material. The fibrous covering was composed of benign proliferation of fibroblastic cells with no mitotic figures. There was no evidence of malignant change and the tumour was considered to be a benign brachial plexus desmoid tumour (Figures 4(a) and 4(b)). After the surgery, the patient did well, remained active, did not complain and had full use of the left hand.

## 3. Discussion

Desmoid tumours commonly involve the anterior abdominal wall and rarely involve the chest wall, axillary, infraclavicular, or supraclavicular areas. Risk factors for the formation of desmoid tumours previously identified include trauma and prior surgical procedures leading to scar formation [11].

Histologically, they are benign, slow growing but are aggressive tumours that can encase or infiltrate the brachial plexus along its course. These lesions are described as well-differentiated fibroblasts or myofibroblasts in an associated abundant collagen matrix with scanty or absent mitotic activity or other atypical cytological features [12]. Differential diagnosis of this tumor type includes fibrosarcoma which metastasizes and nerve sheath tumors such as schwannomas and neurofibromas because of their anatomical location [11].

Evaluation of the brachial plexus presents a great challenge to the clinicians and radiologists. Magnetic resonance (MR) imaging has become the method of choice for evaluating patients with brachial plexopathy [13]. The multiplanar capability of MR imaging and its superior ability to differentiate nerves from vessels and surrounding soft tissues contribute to its success [14]. Magnetic resonance imaging characteristics include a lesion that is hypointense to isointense relative to muscle on T1-weighted imaging, and with a signal intensity similar to fat on T2-weighted images [15]. Imaging is for optimal surgical planning, allowing the surgeon to choose between gross total resection (GTR) and subtotal resection preoperatively.

Gaposchkin et al. in their study emphasise the fact that desmoid tumors present a surgical challenge in that they have a strong tendency for local invasion, surgical margins are poorly delineated, and complete resections are difficult [16]. The mainstay of treatment is a complete resection of the tumour. In the literature, the use of primary or adjuvant radiation therapy to treat desmoid tumors is controversial [17]. Other recommended therapies include systemic chemotherapy with doxorubicin [18]. The use of tamoxifen has been recommended by some authors who noted an increase in the number of estrogen receptors and antiestrogen binding sites in these tumors [19]. Further investigation into the *c-KIT *gene has been proposed, which would determine response to the imatinib mesylate therapy that is used in other gastrointestinal stromal tumors [3].

## 4. Conclusion

Many benign tumors occur along the course of the brachial plexus and involve or impinge on its various components. In our case, the tumor was histologically confirmed to be a desmoid tumor. These tumors are slow-growing, fibrous, nonmalignant lesions with a low propensity for metastasis. Histologically, they are benign, slow growing but can be aggressive encasing or infiltrating the brachial plexus along its course. Imaging with MRI is superior and is for optimal surgical planning, allowing the surgeon to choose between gross total resection (GTR) and subtotal resection preoperatively. Resection is the mainstay of treatment. Residual tumor mass may be treated with radiotherapy or brachytherapy. Further investigation into the c-KIT gene will determine the role of imatinib mesylate therapy in these tumours.
